# Activation of Innate Immune-Response Genes in Little Brown Bats (*Myotis lucifugus*) Infected with the Fungus *Pseudogymnoascus destructans*

**DOI:** 10.1371/journal.pone.0112285

**Published:** 2014-11-12

**Authors:** Noreen Rapin, Kirk Johns, Lauren Martin, Lisa Warnecke, James M. Turner, Trent K. Bollinger, Craig K. R. Willis, Jamie Voyles, Vikram Misra

**Affiliations:** 1 Department of Microbiology, Western College of Veterinary Medicine, University of Saskatchewan, Saskatoon, Saskatchewan, Canada; 2 Department of Biology, University of Winnipeg, Winnipeg, Manitoba, Canada; 3 Department of Pathology, Western College of Veterinary Medicine, University of Saskatchewan, Saskatoon, Saskatchewan, Canada; 4 Department of Biology, New Mexico Tech, Socorro, New Mexico, United States of America; Indiana University, United States of America

## Abstract

Recently bats have been associated with the emergence of diseases, both as reservoirs for several new viral diseases in humans and other animals and, in the northern Americas, as hosts for a devastating fungal disease that threatens to drive several bat species to regional extinction. However, despite these catastrophic events little Information is available on bat defences or how they interact with their pathogens. Even less is known about the response of bats to infection during torpor or long-term hibernation. Using tissue samples collected at the termination of an experiment to explore the pathogenesis of White Nose Syndrome in Little Brown Bats, we determined if hibernating bats infected with the fungus *Pseudogymnoascus destructans* could respond to infection by activating genes responsible for innate immune and stress responses. Lesions due to fungal infection and, in some cases, secondary bacterial infections, were restricted to the skin. However, we were unable to obtain sufficient amounts of RNA from these sites. We therefore examined lungs for response at an epithelial surface not linked to the primary site of infection. We found that bats responded to infection with a significant increase in lungs of transcripts for Cathelicidin (an anti-microbial peptide) as well as the immune modulators tumor necrosis factor alpha and interleukins 10 and 23. In conclusion, hibernating bats can respond to experimental *P. destructans* infection by activating expression of innate immune response genes.

## Introduction

Bats, members of the mammalian order *Chiroptera*, have evolved a range of characteristics that allow them to adapt to changing environmental conditions. They are the only mammals capable of powered flight, most bat species undergo torpor to conserve energy and species that inhabit high northern latitudes hibernate for up to eight months with body temperatures below 10°C [1]. Bats are extremely diverse, making up a fifth of all known mammalian species. They occupy a variety of niches across most of the world where they contribute in many ways to ecological balance [2].

Bats have been implicated as reservoirs for human bacterial [3]–[5] and viral pathogens [6]. In recent years bats have also been associated with the emergence of several devastating viral diseases in humans and other animals [7]–[15]. Circumstantial evidence even suggests that most human and domestic animal pathogens of at least four viral families – *Coronaviridae, Paramyxoviridae, Lyssaviridae* and *Filoviridae* may have arisen in bats [15]–[17] and bats are more likely than rodents to host zoonotic viruses [18]. Interestingly, with the exception of rabies and other *Lyssaviruses*, viruses do not appear to cause overt pathology in bats, suggesting the evolution of benign relationships between bats and their pathogens [14], [19].

Despite the growing realization of the importance of bats in environmental and human health and disease, we know relatively little about how bats interact with their pathogens and commensal microbes. Data on their immune responses to infection during torpor or long-term hibernation are particularly scarce because individuals of some species spend as long as 8 months a year in hibernation (Norquay and Willis, in press) and are therefore difficult to sample. The few studies on bats and other mammals suggest that the energy conserving benefits of torpor and hibernation may be enhanced by a state of immunosuppression [20].

The fungus *Pseudogymnoascus destructans* (formerly known as *Geomyces destructans*) is a cold-adapted saprophytic fungus that targets hibernating bats. It appears to be a recent introduction to the northern Americas [21] and in recent years has been responsible for the death of an estimated 6 million bats and the potential regional extinction of some species [22], [23]. This disease is called White Nose Syndrome (WNS) for the white fungal mycelia on the faces and wings of affected bats. Although damage caused by the fungus is restricted to the superficial skin, infected bats clearly show signs of systemic physiological perturbation such as dehydration, hypovolemia and metabolic acidosis [24]. Infected bats arouse from torpor more frequently than uninfected bats [25], [26] possibly leading to emaciation.

Recently, we investigated the pathogenesis of *P. destructans* [24], [25]. We used samples collected during the experiment to address the question: Can hibernating bats respond to infection by activating genes responsible for innate immune and stress responses? While the site of primary site of fungal infection and occasional secondary bacterial infection was the skin of the wings [25], most of this tissue was needed for histopathology and fungal detection and we were unable to obtain sufficient amounts of RNA from these sites. To test the hypothesis that bats mount a systemic response to infection we examined lungs as an example of an epithelial surface which could have come in contact with aerosolized fungal spores despite being remote from the site of infection or may have responded to infection of peripheral areas [27], [28].

## Materials and Methods

### Tissue Samples

Fifty four Male *M. lucifugus* bats were collected from a WNS-free cave in Manitoba, Canada. Protocols for collecting and transporting bats, infection with *P. destructans*, maintenance of bats in hibernation and sample collection have been described previously [24], [25]. Briefly, bats in groups of 18 were either sham inoculated or inoculated with N. American or European isolates of *P. destructans*. Bats were housed at 7°C and greater than 97% humidity with *ad libitum* water. All bats were equipped with data loggers to monitor skin temperatures. Bats were euthanized during the experiment when humanely required or at the termination of the experiment 120 days after inoculation. Immediately following euthanasia samples from segments of wing as well as various tissues were preserved in RNALater (Life technologies, AM7021) or in formalin. Samples in RNALater were kept at −20°C until they were processed. We did not find *P. destructans* isolate-specific differences and treated the bats as either infected or sham-infected (control).

### Histological classification

During necropsy we collected representative samples for histopathology from all major organ systems. In addition, representative samples were taken from all areas of the wing and rolled on dental wax before placing in 10% neutral buffered formalin. Tissues were processed routinely for histology, 5 µm sections cut and stained with periodic acid-Schiff stain to highlight fungal hyphae. Liver and other tissues were processed routinely and stained with hematoxylin and eosin. Wings were subjectively scored on a scale of 0 to 5 with 5 being very severe with >50% of wing covered in fungal hyphae. Bacterial score was from 0 to 5 with 5 indicating wide-spread and abundant bacteria being present in many areas within the dermis and underlying connective tissues. Interstitial lung neutrophil assessment was similarly evaluated on a scale of 0 to 5, with 5 being very severe.

### RNA Extraction

Tissues were homogenized in 2 ml sealed vials with a 5 mm steel bead, 0.1 g of 0.1 mm zirconium silica beads, 350 µL of RLT buffer (with β-mercaptoethanol) (RNeasy Plus Kit, Qiagen, 74136) using a Retsch MM400 tissue homogenizer at 30 Hz twice for 2 minutes each. RNA from tissues was extracted using the procedure provided with the RNeasy Plus Kit.

### cDNA Synthesis

cDNA synthesis was performed with 1 µg of RNA per reaction using the Quantitect Reverse Transcription Kit (Qiagen, 205313). cDNA samples were either stored at 4°C if proceeding to use in qRT-PCR, or stored at −80°C until they were needed.

### Primers

To identify *Myotis* genes for various cytokines and proteins involved in immune responses we scanned the *M. lucifugus* genome for regions with sequences similar to transcripts of proteins from human, mouse and rat. For genome sequences that were not annotated, *M. lucifugus* exons detected were compared with known exon-intron junctions in corresponding genes in human, mouse and rat to ensure that the sequences did indeed represent homologous genes. For mRNAs where qRT-PCR primers had been described for other species, *M. lucifugus* primers were designed from the sequence of corresponding *M. lucifugus* genes. For mRNAs where such information was not available we designed primers to optimize for annealing temperature and specificity, and to minimize dimer formation. In all cases the veracity of the PCR products was determined by electrophoresis and confirmed by sequencing. Details of primers are in Table 1.

**Table 1:** Primer sequences used for quantitative RT-PCR estimation of *Myotis lucifugus* innate response genes.

Target	Direction	Sequence^a^	Ref^b^	Reference Sequence^c^	Reference Organism^d^
Cathelicidin	Forward	C**G**C**G**GCAG**C**C**C**CC**C**GAGGA**G**TG	[40]	CACAGCAGTCACCAGAGGATTG	Human
	Reverse	G**T**CCTGGT**CC**AGGGT**G**AC**G**		GGCCTGGTTGAGGGTCACT	
Dectin-1	Forward	TGG**CAT**A**CCGATCTG**A**GCT**	[41]	TGGACGAAGATGGATATAC	Rat
	Reverse	AAG*T*CACAG**C**A**A**T**GGA**A		CAAGCACAGGATTCCTA	
Defensin B-1	Forward	ATGAG**GCTC**T**T**C**C**AC**A**T**C**CTGCT	[42]	ATGAGAACTTCCTACCTTCTGCT	Human
	Reverse	CTCTGTA**G**CAG**TA**GCC		CTCTGTAACAGGTGCC	
Hepcidin	Forward	CCTGACCAGTG**T**CTC**A**G**C**T**C**	[43]	CCTGACCAGTGGCTCTGTTT	Human
	Reverse	**GATC** CCCAC**TG**TTTA**GA**AT		CACATCCCACACTTTGATCG	
IL-17A	Forward	AGAGATATCCCTCTGTGATC	[44]	AGAGATATCCCTCTGTGATC	Human
	Reverse	**C** ACCCCAAA**A**TT**G**TCTCAGG		TACCCCAAAGTTATCTCAGG	
IL-23A	Forward	ATGCTGG**GGA**GCAGAG**C**TGT**G**	[45]	ATGCTGGATTGCAGAGCAGTA	Mouse
	Reverse	**GT** GGGG**A**ACAT**C**ATTT**G**T**C**GTCT		ACGGGGCACATTATTTTTAGTCT	
GAPDH	Forward	TGCCTCCTGCACCACCAACTGC	NA	NA	NA
	Reverse	GGGCCATCCACAGTCTTCTGGG	NA	NA	NA
CytB	Forward	CCCCHCCHCAYATYAARCCMGARTGATA	[46]	Identical	Bat
	Reverse	TCRACDGGNTGYCCTCCDATTCATGTTA		Identical	
HSP70	Forward	AGGCCAGCCTGGAGATCGACTCC	NA	NA	Myotis
	Reverse	CACCTTGGGGATGCGGGTGGAGC	NA	NA	
TNFα	Forward	ATGAGCACTGAAAGCATGATC	NA	NA	Myotis
	Reverse	CGATCACCCCCAAGTGCAG	NA	NA	
NOS2	Forward	ATAGAGGAACATCTGGCCAG	NA	NA	Myotis
	Reverse	AAGACCTGCAGGTTGGACCAC	NA	NA	
IL-10	Forward	ATGCCCAGCTCAGCACTGCTC	NA	NA	Myotis
	Reverse	CTATGTAGTTGATGAAGATGTC	NA	NA	
GRP78	Forward	TTTGACCTTGGTGGTGGAACC	NA	NA	Myotis
	Reverse	CTTGGTGTTGAGAAGACAGGGC	NA	NA	

*Myotis* homologues for innate response genes were identified by scanning the *Myotis* genome for sequences that matched those of well-characterized reference organisms. Where qRT-PCR primers for reference organisms^d^ had been validated in the literature^b,c^, we used the corresponding *Myotis* sequences as primers. *Myotis*-specific substitutions in these primers are in bold^a^. For other genes (HSP70, TNFα, NOS2, IL-10, GRP78) *Myotis*-specific primers were designed from potential *Myotis* exon sequences using parameters for optimal use in PCR reactions. In all cases the identity of PCR products were confirmed by sequencing. Primers were used in qRT-PCR at a final concentration of 1 µM.

### Quantitative Real-Time PCR (qRT-PCR)

Stratagene's MX3005P PCR Cycler was used in conjunction with QuantiFast SYBR Green PCR kit (Qiagen, 204056). In addition to the primer sets mentioned, *GAPDH* and *Cytochrome B* were both used for every sample as normalizers. For each sample tested, 2 µL (1 µM, final concentration) of diluted forward primer, 2 µL (1 µM) of diluted reverse primer, 12.5 µL of SYBR Green Master Mix, and 8.5 µL of cDNA was used. Samples were tested in duplicate in each run and values used in the analysis were averages of the duplicates. The thermal profile used was as follows: 95°C for 5 minutes for initiation, then 40 cycles of 95°C for denaturing for 10 seconds, and 60°C for annealing and extension for 30 seconds with readings at the end of every cycle, then 1 cycle of 95°C for 1 minute, 55°C for 30 seconds, and 95°C for 30 seconds. The denaturation characteristics of the products were determined by incremental increase from 55°C to 95°C. A no template control (NTC) was used in every sample tested. Reactions that generated primer dimers or products with spurious denaturation profiles were not considered. In all cases the identity of the PCR products was confirmed by determining their nucleotide sequences (Macrogen). Only results from reactions that yielded unambiguous results (products of the expected size and sequence, homogenous denaturation profiles, no products for no-template controls) were used for analysis.

### Statistical analysis

Data from qRT-PCR and histopathological scores were analysed with SPSS Statistics version 21. For qRT-PCR the relative levels of a transcript for each bat were calculated as Cycle threshold (Ct) normalized separately (ΔCt) for levels of transcripts for two “house-keeping” genes – Glycerol 6 phosphate dehydrogenase (GAPDH) and Cytochrome B. A lower Ct (or ΔCt) of one (1) indicates approximately a two-fold higher concentration of RNA. The significance of differences of mean values of ΔCt between infected and mock-infected (control) bats were determined using a student T-test. Pearson's coefficients were calculated for the ΔCt levels for cytokine transcripts for bats in each treatment class and lung interstitial neutrophil scores and mean bacterial and hyphae scores for 5 wing sections for each bat.

### Ethics statement

Methods were approved by the University of Saskatchewan committees on Animal Care (Protocol #20100120) and Biosafety (Permit #VMB03).

## Results

Recently we demonstrated that the European and North American strains of *P. destructans* were equally pathogenic for hibernating *M. lucifugus* [24], [25]. During these experiments histological sections from wings and other tissues were examined and graded for the presence of hyphae, edema, necrosis, bacteria (epidermal or invasive), neutrophil infiltration and inflammation. We also preserved samples which were then used in this study.

**Figure 1:**
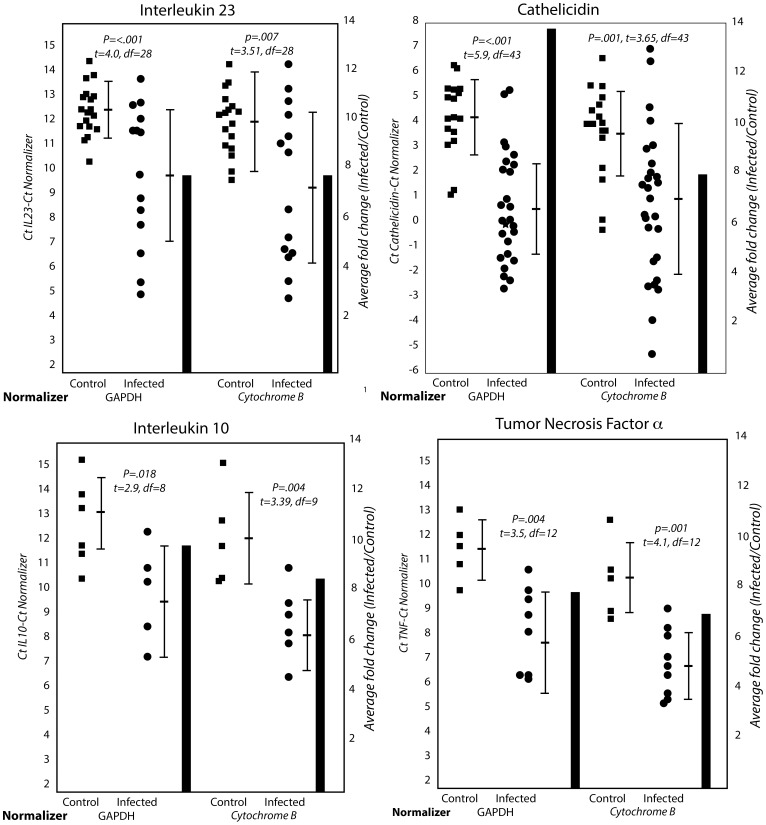
Differences in levels of transcripts for IL23, IL10, Cathelicidin and TNFα in lungs of hibernating mock-infected (control) or *P. destructans*-infected bats. Each square (control) or dot (infected) represents the value from a bat. The relative levels of a transcript for each bat are depicted as Cycle threshold (Ct) normalized separately (ΔCt) for levels of transcripts for two “house-keeping genes – Glycerol 6 phosphate dehydrogenase (GAPDH) and Cytochrome B. The horizontal bar represents the mean while the vertical bar indicates standard deviation from the mean. Significance (P), test statistics (t) and degrees of freedom (df) are indicated. A lower value of one (1) ΔCt (or Ct) indicates approximately a two-fold higher concentration of RNA. Infected bats as a group had lower ΔCt values for the four genes. The histograms represent fold differences between infected and control bats. Only results from reactions that yielded unambiguous results (products of the expected size and sequence, homogenous denaturation profiles, no products for no-template controls) were used for analysis. The number of results that fitted these criteria for each analysis were: IL23 - Control  = 17, Infected  = 13; Cathelicidin – Control  = 17, Infected  = 28; IL10 – Control  = 5, Infected  = 6; TNFα – Control  = 5, Infected  = 9.

We determined levels of transcripts for several immune and stress response genes (Table 1) in lungs from infected and control bats. Only those qRT-PCR reactions that yielded unambiguous results were considered. There were significant differences for transcripts for the interleukins (IL) 10 and 23, the pro-inflammatory cytokine tumour necrosis factor alpha (TNFα), and the anti microbial and immunoregulatory peptide Cathelicidin (Figure 1). In Figure 1a lower ΔCt of one indicates approximately a two-fold higher concentration of RNA. Infected bats as a group had lower ΔCt values (on an average 8 to 14 fold higher concentrations of RNA, histograms in Figure 1) for the four genes.

While the group of infected hibernating bats had higher levels of transcripts for IL10, IL23, Cathelicidin and TNFα than hibernating uninfected bats, the levels varied over a wide range with some infected bats having levels comparable to (or lower than) those of uninfected animals. Since the level of fungal infection and pathology varied considerably among infected bats, we attempted to correlate transcript levels with scores for fungal infection, fungal hyphae and bacterial infection in wings and neutrophil accumulation in lungs (Table 2). IL23 and Cathlicidin levels correlated with all four parameters of disease and inflammation. IL10 correlated with level of infection, and hyphae and bacterial scores in the wing, and TNFα correlated with level of infection and wing hyphae score. There was an inverse relationship between transcript levels and ΔCt. The correlation coefficients are therefore negative.

**Table 2:** Correlation of activation of innate response genes with fungal and bacterial infection, and neutrophils in the lung interstitium, and fungal hyphae and bacterial scores in wing tissues.

Correlate		IL23		Cathelicidin		IL10		TNF	
		GAPDH	CytB	GAPDH	CytB	GAPDH	CytB	GAPDH	CytB
Infection	Correlation (r)	−0.60	−0.55	−0.67	−0.49	−0.72	−0.75	−0.71	−0.77
	Significance (P)	<0.001	0.001	<0.001	0.001	0.018	0.008	0.004	0.001
	N	30	30	45	45	10	11	14	14
Ave. hyphae score	Correlation (r)	−0.67	−0.60	−0.64	−0.42	−0.71	−0.69	−0.57	−0.60
	Significance (P)	<0.001	<0.001	<0.001	0.004	0.02	0.018	0.033	0.024
	N	30	30	45	45	10	11	14	14
									
Ave. bacterial score	Correlation (r)	−0.77	−0.65	−0.61	−0.42	−0.69	−0.61		
	Significance (P)	<0.001	<0.001	<0.001	0.004	0.026	0.046	NS	NS
	N	30	30	45	45	10	11	14	14
Lung Interstitial neutrophil score	Correlation (r)	−0.65	−0.64	−0.35	−0.45				
	Significance (P)	0.001	0.001	0.036	0.005	NS	NS	NS	NS
	N	22	22	37	37	10	11	14	14
									

Pearson's coefficients were calculated for the ΔCt levels for cytokine transcripts for bats in each treatment class and lung interstitial neutrophil scores and mean bacterial and hyphae scores for 5 wing sections for each bat.

N =  number of samples with usable Ct values. NS = not significant.

## Discussion

Our results suggest that hibernating *M. lucifugus* are capable of inducing the expression of genes for cytokines in response to infection. The bats we examined were kept under conditions that closely mimicked temperature and humidity encountered in their natural hibernacula (i.e., 7°C and >97% relative humidity) [24], [25]. During hibernation *M. lucifugus* body temperature is maintained within 1–2°C of ambient temperature for weeks at a time with return to normothermic temperatures during periodic arousal [24], [25]. It is difficult to imagine mammalian gene expression at temperatures this low and it is possible that the genes were transcribed during the arousal periods. Our previous study indicated that *P. destructans*–infected bats aroused more frequently than uninfected bats [25], a pattern which has also been observed in the wild [26] and this may also have contributed to higher basal levels of gene expression in their cells. However, our observation that the levels for IL 23, Cathelicidin, IL 10 and TNFα and not the other transcripts examined (Dectin 1, Defensin B1, Hepcidin, IL 17, Cytchrome B, heat shock protein 70, nitrogen oxide synthetase) were higher in the lungs of infected bats, suggest that the response was a specific to infection.

We observed mild to moderate levels of interstitial neutrophils in the lung of infected bats. On average, the lungs contained 8 to 20 times more transcripts for the anti-microbial peptide Cathelicidin as well as for the immune modulators TNFα and IL 10 and 23. The increase in levels of IL 23, Cathelicidin and IL10 correlated with the lung interstitial scores (Table 2). Little is known about the role in bats of these bioactive proteins, information from other species indicates that they are intimately involved in the response to infection. For instance:

Cathelicidins belong to a family of antimicrobial peptides with an intrinsic ability to kill bacteria and fungi but they also function as chemoattractants for inflammatory cell recruitment and cytokine release. They are found in the lysosomes of neutrophils, macrophages and epithelial cells after activation or infection with a wide variety of pathogens [29]. Humans, mice and dogs have a single gene for Cathelicidin while several related genes have been discovered in pigs, horses, cattle and chickens. *M. lucifugus* has at least two genes with similarity to the human gene. Our primers were designed to amplify transcripts for the gene most similar to that for human Cathelicidin.

Interleukin 23 is a multifunctional pro-inflammatory cytokine involved in both innate and adaptive responses [30]. It is an important mediator of Dectin-dependent response of the protective neutrophils to lung infections by *Aspergillus* [31]. It is also important in the maturation of T helper cells. In addition to being protective IL23 may also play a negative role by inducing chronic inflammation and exacerbating the effects of *Aspergillus* and *Candida* infections [32].

Interleukin 10 is also known as cytokine synthesis inhibitory factor (CSIF) because of its role in modulating the inflammatory response. Its effect is to suppress Th1 responses and stimulate Th2 responses leading to B-cell survival and proliferation and antibody synthesis. IL10 is produced by a number of cell types including monocytes, and activated subsets of regulatory and helper T-cells B-cells. Its synthesis is tightly regulated and is mediated by the NFκB and AP1 pathways after stimulation by commensal and pathogenic microorganisms [33]. If the role of IL10 in WNS is indeed to direct the immune system to a Th2 response, our results are in contrast with those of Moore et. al. [34] who found increased levels of IL4 in the serum of WNS-affected bats. Work in other species suggests that IL4 directs the immune system to a Th1 response.

Tumour necrosis factor alpha (TNFα) is a monocyte derived cytotoxin implicated in tumour regression, septic shock and cachexia. TNFα binding to its receptor stimulates the activation of the transcription factor, NFκB, which activates gene expression required for several defensive responses [35].

The presence of neutrophilia in the lung interstitia of infected bats, despite a lack of obvious pathology or the presence of microorganisms, is puzzling. However, these observations may be explained by: a) Injury in the skin can lead to complement-induced TNFα expression in the lungs followed by inflammation and neutrophil infiltration [27]. b) Immune responses at mucosal surfaces are coordinated, with stimulation at one surface leading to responses at other mucosae [36]. The pulmonary response may have been due to stimulation of the upper respiratory tract by fungal spores. c) There have been suggestions that hibernating mammals show marked leukopenia [20] raising the possibility that circulating leukocytes are sequestered in some organ, possibly the lungs. The infected animals may have responded to inflammation in the wings with an increase in neutrophils, which then migrated to the lungs. d) Lung neutrophils in infected bats, and the accompanying increase in the transcripts of the immune modulators may represent the end of the infectious process in the lungs and the removal of fungal spores and hyphae. This suggests that bats are capable of suppressing a lung or systemic infection by *P. destructans* and may explain why WNS, the disease, occurs only after bat-to-bat contact rather than by infection through inhalation.

While relatively little is known about the response of bats to infections, work with other species indicates that the host's response to pathogens is complex involving the interconnected innate, relatively non-specific mechanisms as well as the more specific adaptive systems of humoral and cellular immunity. The increased transcripts we detected in infected bats represent but a few components of this complex network.

While our study shows that hibernating bats can respond to an infection, it does have some limitations and our results should be interpreted with caution. For instance, we have used increase in the stable levels of transcripts for various cytokine genes as a surrogate for increases in their respective proteins. However mRNA and protein levels do not always correlate, largely because of complex regulatory processes that separate transcription of mRNAs from their translation into protein [37]–[39]. In addition, only one of the transcripts, for Cathelicidin, is directly linked to an anti-fungal response. The remainder, IL 23, IL 10 and TNFα may be indirectly related or may represent a non-specific response to the stress of infection.
